# Photocatalytic aerobic oxidative functionalization (PAOF) reaction of benzyl alcohols by GO-MIL-100(Fe) composite in glycerol/K_2_CO_3_ deep eutectic solvent

**DOI:** 10.1038/s41598-022-22369-9

**Published:** 2022-10-29

**Authors:** Sepideh Abbasi, Mohammad Reza Naimi-Jamal, Shahrzad Javanshir, Akbar Heydari

**Affiliations:** 1 Research Laboratory of Green Organic Synthesis and Polymers, Department of Chemistry, Iran University of Science and Technology (IUST), Tehran, 16846-13114 Iran; 2 Pharmaceutical and Heterocyclic Compounds Research Laboratory, Department of Chemistry, Iran University of Science and Technology, Tehran, 16846-13114 Iran; 3 Chemistry Department, Tarbiat Modares University, Tehran, 14155-4838 Iran

**Keywords:** Chemistry, Catalysis, Organic chemistry

## Abstract

An MIL-100 (Fe)/graphene oxide (GO) hybrid, a fairly-known composite, was made through a simple one-step procedure and played a highlighted role in the photo-induced oxidative functionalization of the benzylic C–H bond. To identify the given binary composite, various techniques were applied: FT-IR, P-XRD, SEM, nitrogen absorption–desorption analysis, TGA, TEM, and UV–Visible DRS spectra. Proportions of GO used within the structure of the prepared composite differently ranged from low to high amount, and the most optimized ratio met at 38.5% of GO as the most efficient catalyst. Additionally, the reaction ran in Glycerol/K_2_CO_3_ (2:1) as the optimal solvent. The elemental roles of O_2_^**·**−^ and OH^−^ were supposed to be the major ones for running a tandem oxidation-Knoevenagel reaction. The heterogeneity and reusability of the catalyst were also examined and confirmed after five successive runs.

## Introduction

Green chemistry and its subunits have continuously focused on developing processes based on renewable energy sources^1, 2^. Photocatalytic methods based on visible light have a special place in green strategies since they are susceptible to solar energy^3, 4^. Chemical researchers have been attracted to photocatalysis in synthetic organic chemistry in favor of its conduct toward the highest standards^5, 6^. Nevertheless, despite its high potential, challenges such as the short-lived photo-generated electron–hole pairs prevent the widespread application of photocatalytic technology^7^.

In recent years, many semiconductor materials, especially species with conjugate structures, have been extensively studied in optical instruments^7, 8^. Among them, 2D transition metal chalcogenides^9^, conductive polymers^10^, and carbon-based backbones such as carbon nitride^11^, carbon nanotubes^12, 13^, graphene^14^, and graphene oxide (GO)^15^ are of particular interest. The development and improvement of economical and cost-effective methods for GO preparation, as a promising option, is more considered than the others^16, 17^. In addition to the widespread application in electronic and optical tools, their extended π-conjugated systems are employed in chemical transformations. Applied visible wavelength (up to 455 nm), high thermal and chemical stability, and the presence of surface functional groups are the major characteristics that not only allow interaction with different species but also could reduce the need for additional procedures of functionalization^18, 19^. The short lifetime and low quantum efficiency of photo-induced electron–hole pairs make pure GO a less suitable photocatalyst^20–22^. To modify such deficiencies, composite formation with other metals and semiconductors, crystal structure modification, (hetero)atom-doping, and nano-based surface decoration with various structures are the actions taken on pure GO. Among the given strategies, band-gap engineering has put on the (hetero)atom-doping and nano-based surface decoration as the few most effective ways to improve the photocatalytic activity of graphene oxide. Several approaches will be able to enhance GO performance. Introducing atomic impurities, as a sufficient measure, can expand the light absorption region, modify the band-gap position, and defer the electron–hole recombination.

Besides, surface decorating of GO using various semiconductors to improve the charge transfer in the interface can also improve the performance of both components. Jain et al. used a GO-based photocatalytic system coated with zinc oxide (ZnO) nanoparticles for water treatment through the Fenton process^23^. Compared to its intact, primary components, the finalized composite has significantly improved the durability of the donor–acceptor pairs and interactions with reactive species.

As the most attractive porous materials, metal–organic frameworks (MOFs) have captured incredible attention in the last decade. Unique features such as purposeful and programmable architecture, structural diversity, large surface area, applicability in chemical sensing, separation, gas absorption and storage, and, most importantly, catalysis have multiplied the importance of these frameworks^24–27^. However, the bearing fruit use of MOF-based optical elements and devices has been (hampered) limited by disadvantages such as low light absorption and high charge-transfer resistance. Specifically, the magic starts combining the untouched components. In the realm of our magic, chemistry, and everything cannot be impossible. Feasibility could depict itself in the combination of MOFs and materials with desirable properties to reduce their demerits and increase their merits^28, 29^.

The development of catalytic methods has been able to find an irreplaceable place in synthetic organic chemistry for the removal of the negative impact rooted in human populations and their peripheral needs. Developing multifunctional catalytic systems for successive cascade transformations to produce complex organic molecules is a highly creative response to these emerging must-haves^30^. Based on the proven catalytic properties of MOFs and the optical features of GO, they are capable of joining together as those porous and multi-π-stacking surfaces were exploited for photocatalytic systems work mutualto ly by another structure^31, 32^. Graphene and Graphene-derived structures have proven to be excellent photocatalysts for oxidation and reduction reactions, for example in photochemical transformation of CO_2_ into different organic structures, splitting of H_2_O, removal of formaldehyde and NO_x_ pollutants, or photocatalytic degradation of dyes^33–35^.

For this current work, the Fe-MOF/graphene oxide (Fe-MOF/GO) binary composite was prepared using a simple and economical method under ultrasonic irradiation. The prepared composite was scanned structurally using different characterization techniques, and the inter-relationship between the chemical, physical and optical properties of the composite and its function was confirmed. We did the required experiments to reach the optimal destination where we could run a cascade aerobic oxidative functionalization (AOF) reaction at that and could go forwards to the next step. Further results indicated the superb performance of the prepared photocatalytic system under optimized conditions to perform this reaction on different derivatives. Additionally, the observation of mechanistic studies revealed an increase in visible light absorption and charge transfer by the prepared photocatalytic system and a decrease in electron–hole pairs recombination. The latter output is as necessary as the former one to have efficient photocatalysis. Examination of the stability and recovery of the prepared composite showed its extraordinary stability during the process.

## Experimental

### Materials and instruments

All preparatory materials, as a professional chemist’s routine, were supplied from prestigious companies and utilized without any need for purification. The as-prepared composite was also analyzed by various identification techniques, clarified as follows: to perform FT–IR spectroscopy, the NICOLET IR100 apparatus was used and carried out in the 400–4000 cm^–1^ region. Thermogravimetric analysis (TGA) of catalyst was obtained via the NETZSCH apparatus, which was run from room temperature to 600 °C with a heating speed of 10 °C per minute. Powder X-ray diffraction pattern was recorded by a Philips X'pert 1710 diffractometer at room temperature using Cu-Kα (α = 1.54056 Å) via charted Bragg–Brentano geometry over the 2θ of 10°–60°. To perform UV–Visible DRS spectroscopy, an MPC-2200 spectrophotometer was utilized. SEM scanning was done by Philips XL 30 and S-4160 devices with a gold-aided coating laid out on nanoparticles within the EDX test. TEM images were taken at 120 kV by Philips apparatus (model CM120). The BET analysis measured N_2_ adsorption at 77 K using a NOVA4000 version of Quanta chrome. An Echrom GC-FID (Agilent; A90) gas chromatograph was used to analyze the products quantitatively. The source of the applied light was a Xenon lamp (500 W; Xe source) whose lighting characteristics are as follows: (1) white light arrays, (2) it includes all UV–Visible light wavelength ranges of 200 nm to ca. 1000 nm, with a luminance of around 35–50 lumens/watt from the 40-cm site of reaction (according to its production company’s data).

### GO-MIL-100(Fe) composite formation procedure

Initially, the addition of 1,3,5-benzene tricarboxylic acid (H_3_BTC) (50 mg, 0.238 mmol) to 1.2 mL of diluted ethanol (using an equal volume of DI water) resulted in a solution that was immediately shaken by ultrasonic irradiation for 30 min to disperse the component homogeneously. Then, GO (prepared through Hummer’s method^36^) in various weight percentages of 5, 10, 20, 50, 100, 150, and 200 wt. % were added to 1.2 mL of Fe(NO_3_)_3_·9H_2_O (50 mg, 0.25 mmol) aqueous solution. Then, the subsequent mixture was stirred for 10 min. Next, the two prepared solutions were mixed and stirred for another 10 min. Afterward, Et_3_N (0.05 mL) was added to this solution and allowed to be swiftly mixed with the former mixture at 60 °C for 24 h. In the end, the reaction mixture passed a centrifugation step (by 98% EtOH) to eliminate the impurities or any unreacted monomers. The obtained composite was finally dried up at 60 °C overnight.

### General catalytic procedure

A specific amount of the catalyst (GO-MIL-100(Fe); 5 mg) was mixed with 2 mL of the aimed alkaline deep eutectic solvent (DES) Glycerol/K_2_CO_3_ (with a ratio of 2:1 respectively). The container of reaction was put into a photocatalytic set (within the 40-cm exposure distance of the lighting source). To this viscous potion, benzylic alcohol (1 mmol) was gradually added at room temperature. Running of the desired reaction mixture was done by stirring the mixture at the same temperature under the air bubbling (O_2_ source) for 3 h. The Progress of the oxidative step was continually assessed by thin layer chromatography (TLC) and then GC. Following bubble stoppage, in the next step, malononitrile (1 mmol) was added and the product of the Knoevenagel reaction was checked by TLC. After completion of the reaction, the products were purified by re-crystallization. All the products were characterized by a comparison of their spectral data/melting points with the previously published works in the literature^37, 38^.

## Results and discussion

### Structural analyses

**Figure 1:**
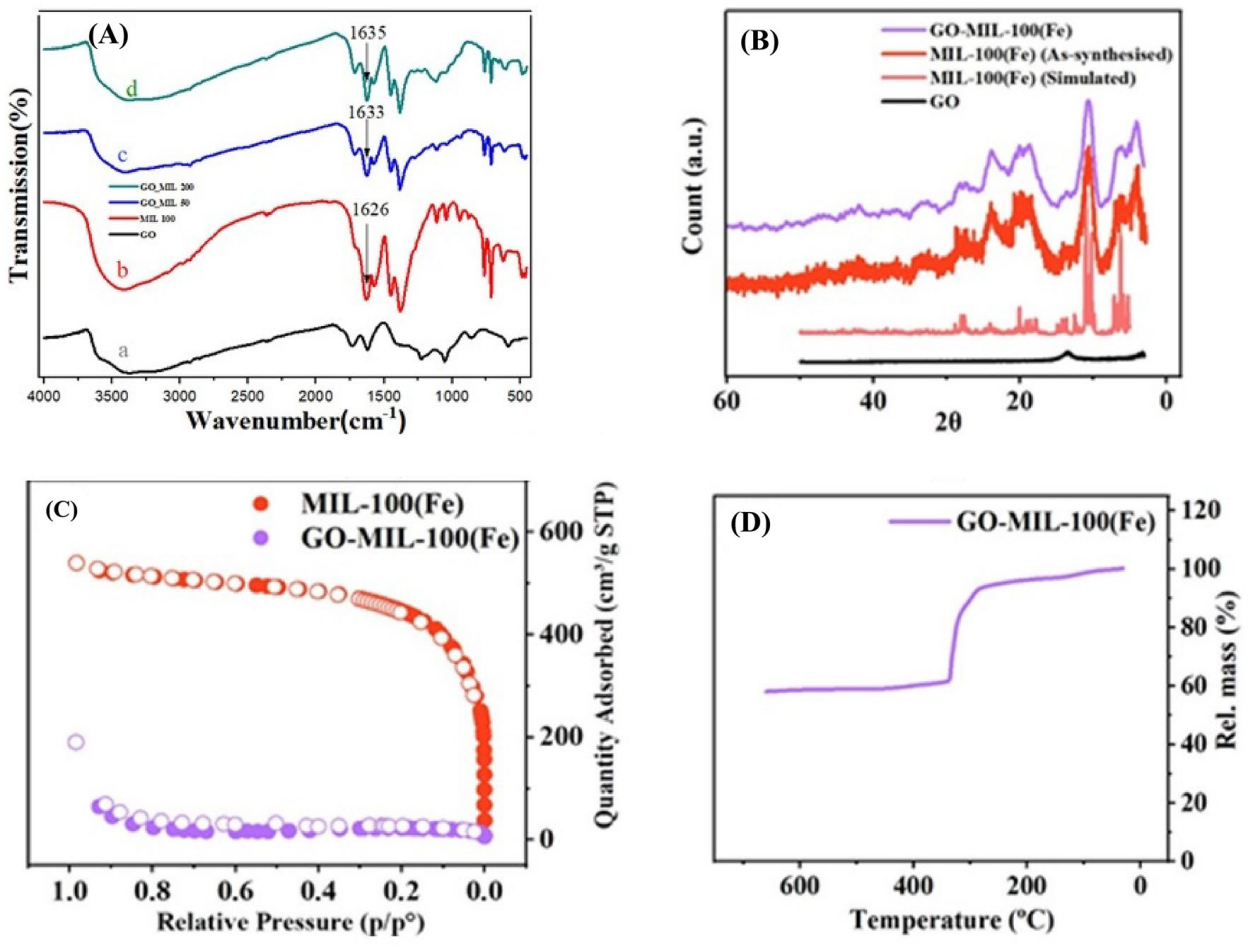
(**A**) FT-IR spectra, pure GO (a), pure MIL-100(Fe) (b), GO (50%)/MIL-100(Fe) composite (c), and GO (200%)/MIL-100(Fe) composite (d), (**B**) PXRD, (**C**) Nitrogen adsorption–desorption isotherms, (**D**) TGA patterns of GO-MIL-100(Fe).

To realize whether the designed composite has been prepared successfully or not, FT-IR spectroscopy was utilized. Given in Fig. 1A, the FT-IR spectra of intact forms of GO, MIL-100(Fe), and GO-MIL-100(Fe) (GO 50% and 200%) hybrids are observed. First of all, there is a broad signal detected in the 3000–3500 cm^−1^ range in the spectra of four prepared samples, showing the hydroxyl groups belong to absorbed water molecules^39^. As shown in Fig. 1Ab, the bands at 1626, 1575, and 1387 cm^−1^ evidence the presence of –COOFe metallic esters in these compounds, and are attributed to the splitting of the coordinated –CO_2_^−^ vibrations^40, 41^. As known, the changes in the FT-IR spectra in composites can be negligible in some circumstances, especially when the intensities of the peaks of one component are much lower than the other one. In our case, the intensity of different peaks in GO is much lower than those in the polar MOF and this may result in overlapping the peaks of GO with the peaks of MOF. In order to show the differences, by increasing the amount of GO to 50% and 200%, the vibration frequency of COO^−^ in the MOF has been moved from 1626 cm^−1^ (Fig. 1Ab, MOF) to 1633 cm^−1^ (Fig. 1Ac, 50% GO) and finally to 1635 cm^−1^ (Fig. 1Ad, 200% GO).

It has been understood that our produced GO is not reduced because of the presence of –OH, –COOH, and epoxide elements of –C–O–C– vibrational frequencies^42^. As evidenced in Fig. 1A, the asymmetric stretching of COO stretching and C–H deformation oscillations were blocked in the two uppermost lines, which was more relevant with the GO adding. These records showed the successful decoration of GO nano-sheets merged with Fe-MOF nanoparticles. Since the low concentration of graphene oxide has been used in the composite, its correlated peaks have become weakened in the spectrum of GO-MIL-100(Fe).

Via a short glance at Fig. 1B, the main differences in PXRD patterns of pure GO, pure MIL-100(Fe), and synthesized GO-MIL-100(Fe) nanocomposite are detectable. The pink-colored XRD pattern of MIL-100(Fe) matches the simulated one reported in previous literature, showing the complete formation of the Fe-based MOF. In the following, it is evident that almost all the characteristic peaks of MIL-100(Fe) have appeared in the pattern of the prepared composite, while no distinguishable broad peak related to GO (at about 9.3° on the black line) is observed. Indeed, under ultrasonication, GO nanosheets become more exfoliated and smaller in size, and more importantly, restricted concertation of GO is used in the preparation of the composite.

Gaining information about the surface area and porosity of the prepared samples is done by Brunauer–Emmett–Teller (BET) analysis. The BET plot of MIL-100(Fe) illustrated the combination of both type I and IV isotherms and huge uptakes of nitrogen gas (N_2_) at low proportional pressure (P/P_0_ < 0.1), which indicates its mesoporous nature formed on a micro-scale (Fig. 1C)^43^. On the other hand, GO-MIL-100(Fe) showed type II isotherm, indicating the non-porous structure of the prepared composite owing to the introduction of GO nanosheets as macropores in the composite (Fig. 1C). Furthermore, according to IUPAC classification, the isotherm of the developed composite showed an H_3_-type hysteresis loop that means the existence of sandwich-like pores in the 2-D network. In more detail, MIL-100 octahedrons composite was desirably trapped among GO nanosheets^44, 45^. Figure 1C further showed enlarged meso-cages. Both isotherm observation and statistic data (Table 1) showed that what is called the specific surface area has become lower in GO-MIL-100(Fe) compared with primary MIL-100(Fe), which now led to the formation of meso-macropores. The pore texture analysis illustrated that the in-situ growth method might make the GO-MIL-100(Fe) composite prone to extra application beyond the synthesis; it may be able to show enough adsorption performance, as well.

**Table 1:** Specific surface areas and micropore volumes of three studied species: GO, MIL-100(Fe), and GO-MIL-100(Fe).

Sample	S_BET_ (m^2^ g^−1^)
GO	83
MIL-100 (Fe)	1121.76
GO-MIL-100 (Fe)	191.84

The stability of the developed GO-MIL-100(Fe) composite against temperature was screened via TG analysis. As displayed in Fig. 1D, the composite showed two main weight loss stages in the 25–600 °C temperature range. The first weight loss occurred below 150 °C, attributed to the exit of solvent molecules from the pores through evaporation. Notably, at 150 to nearly 300 °C, almost no weight loss was observed, which indicates the applicability of the composite up to 300 °C. In the second step, a sharp weight loss happened, starting at 300 °C, and a majority of this weight loss can be due to the degradation of organic ligands laid into Fe-based MOF. Nevertheless, a slight amount of this weight loss is related to the decomposition of functional groups that happened in the GO structure, which may be because of their low content in the composite structure as well as the interactions of these functional groups embedded in GO with the open metal sites in MIL-100(Fe)^46^.

**Figure 2:**
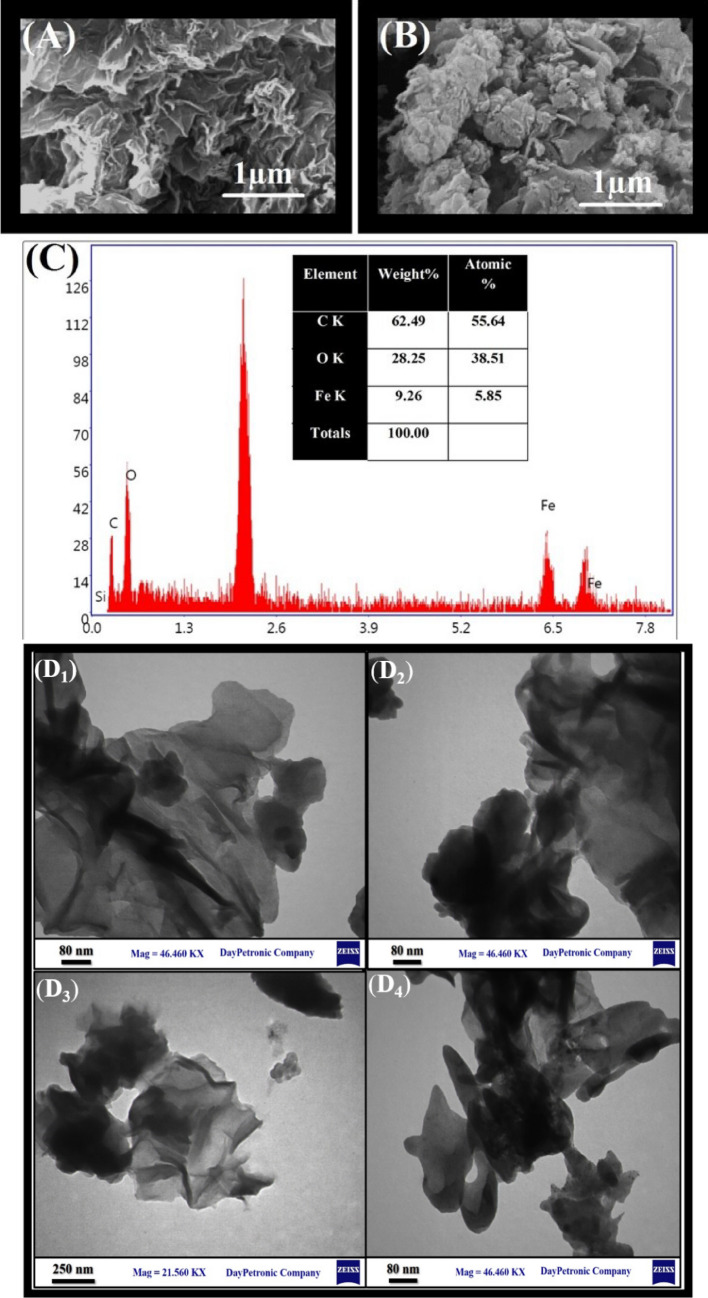
SEM images of graphene oxide (**A**), GO-MIL-100(Fe) (**B**), EDX elemental analysis of GO-MIL-100(Fe) (**C**), and TEM images of GO-MIL-100(Fe) (**D**_**1**_–**D**_**4**_).

The SEM measurements were carried out to evaluate the facial features of GO and the fabricated GO-MIL-100(Fe) composite depicted in Fig. 2A,B. This figure shows that GO sheets have a spiral and crinkled morphology, while MIL-100(Fe) nanoparticles show a unique octahedral morphology with a random connection to the surface of GO sheets. Energy dispersive X-ray spectroscopy (EDX) analysis is applied to identify the elemental composition of GO-MIL-100(Fe), where the presence of C, O, and Fe was certain, as well as confirming its chemical purity (Fig. 2C).

TEM imaging could clarify the porous and layered structure of GO-MIL-100(Fe) composite (Fig. 2D_1_–D_4_). As can be seen in the presented TEM images of GO-MIL-100 (Fe) in different magnifications, MIL-100 (Fe) particles were dispersed on the GO layers. The layered structure with the wrinkled surface belongs to GO typical morphology which contained the distributed MIL-100 (Fe) particles on the GO layers owing to the strong interaction between the MIL-100 (Fe) particles and the GO nano-layers in this structure.

**Figure 3:**
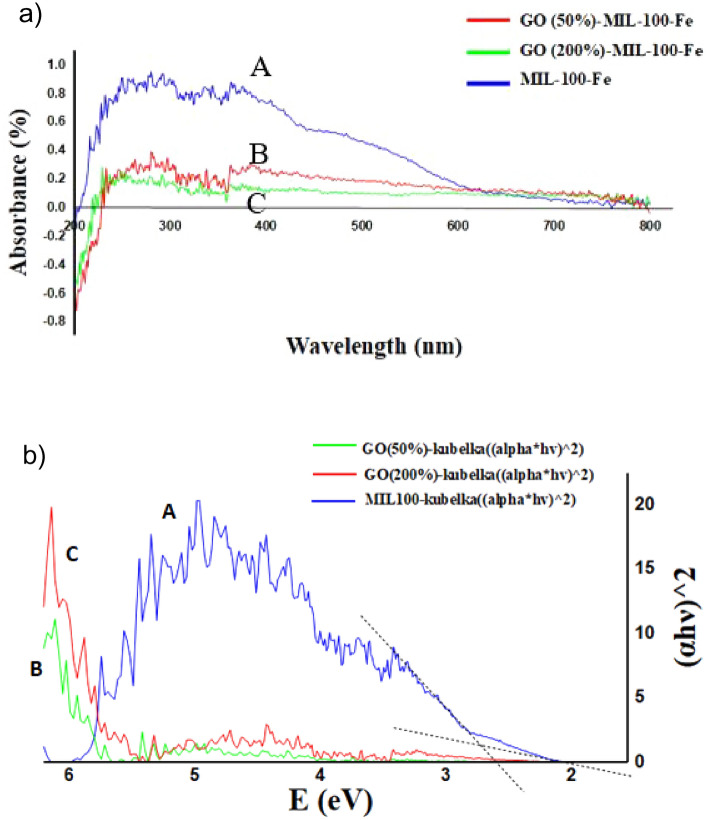
(**a**) Comparable DRS Spectra of pure MIL-100(Fe) (A), 50% Composite (B), and 200% Composite (C). (**b**) Kubelka–Munk plot of pure MIL-100(Fe) (A), 50% Composite (B), and 200% Composite (C).

UV–Vis diffuse reflectance spectroscopy (DRS) technique and the related Kubelka Munk plot of the developed GO-MIL-100(Fe) have been shown in Fig. 3a,b. In Fig. 3a, the UV–Vis DRS spectrum of GO-MIL-100(Fe) shows several absorption signals in the UV region, which mostly is related to the π → π^*^ electron transfer in trimesic acid, indicating the existence of an Fe-based complexed network within the composite. However, the intensity of light absorption in the visible light range is noticeably lower than the one in the UV region, maybe due to the presence of a slight amount of GO in the composite structure. Moreover, in order to calculate the band gap of the prepared composite, the following equation reported by Butler was used^47^:

$$\upalpha {\text{h}}\upnu = {\text{ A}}\left( {{\text{h}}\upnu - {\text{E}}_{{\text{g}}} } \right)^{{{\text{n}}/{2}}}$$

in which E_g_, α, h, and ν are named band gap energy, absorption coefficient, Planck’s constant, and light frequency, respectively. The value of n also depends on the type of transition (n = 1 for direct transition and n = 4 for indirect transition). As Fig. 3b displays, the E_g_ of the GO-MIL-100(Fe) (GO 50%) composite is obtained by drawing the plot of (αhν)^2^ versus energy (hν), giving the value of 2.078 eV (Fig. 3b). The more GO, the lower the band gap will become. So, the combination of GO (50%) and MIL-100(Fe) proves more primary hypothesis that a possible connection has been π–π interaction^48^ among their components.

Compared with many similar papers discussing composites, the more GO in GO-MIL-100(Fe) composites, the lower place for every related curve could be seen. By a similar interpretation, the DRS spectrum of MIL-100(Fe) (A) is compared with the prepared catalysts’ DRS diagrams (B: 50% GO and C: 200% GO). According to the given plot (Fig. 3a), when GO nano-layers were mixed with MIL-100(Fe) nanoparticles, the band gap value of MIL-100(Fe) changed to a lower absorbance. By adding more GO (in percentage) to MIL-100(Fe), we had a substantial fall in both the composites’ DRS spectra.

### Catalytic activity study

**Figure 4:**
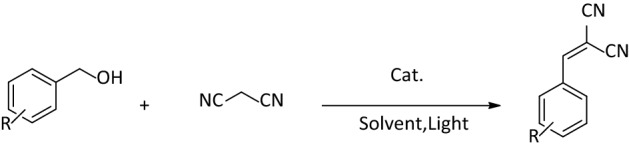
Model reaction for photocatalytic oxidative condensation reaction.

We have examined whether the innovative catalyst can handle multi-stage reactions efficiently by using benzyl alcohols in a photocatalytic oxidative functionalization reaction using air bubbling (Fig. 4).

Every model reaction is up to examine the capability of a reagent or any motivator (catalyst), which, here, we aimed at the commonplace oxidation coupled with the Knoevenagel condensation. The effect of the GO ratio in the presence of 5 mg of composites in EtOH at a definite time was the first assessment. Based on the obtained results, it was understood that among the prepared composites, the one with the ratio of 50% GO yielded the highest efficiency compared to other composites. In fact, from this ratio on, the increase in the content of GO reduces the efficiency of reaction, maybe because more catalytic sites are covered by extra GO sheets.

Further optimizations were performed to investigate the effect of some parameters, such as the amount of the catalyst, time of reaction, temperature, and solvent. As illustrated in Table 2, no product was obtained in the absence of both catalyst and light (Table 2, Entry 1). In addition, the constitutive discrete components of the composite and Fe (NO_3_)_3_·9H_2_O were surveyed (Table 2, Entries 2–4), giving the desired product in low yield. All in all, for this part, the model reaction received support from GO-MIL-100(Fe), showing an increased yield of 57% (Table 2, Entry 5).

**Table 2:** Optimization track of the catalysts used in the PAOF.

Entry	Catalyst	Catalyst (mg)	Solvent	Temp. (°C)	Yield (%)
1	–	–	EtOH	rt	–
2	GO	5	EtOH	rt	18
3	MIL-100(Fe)	5	EtOH	rt	12
4	Fe (NO_3_)_3_·9H_2_O	5	EtOH	rt	15
5	GO-MIL-100(Fe	5	EtOH	rt	57
6	GO-MIL-100(Fe)	5	*n*-Hexane	rt	32
7	GO-MIL-100(Fe)	5	Acetonitrile	rt	18
8	GO-MIL-100(Fe)	5	MeOH	rt	17
9	GO-MIL-100(Fe)	5	H_2_O	rt	13
10	GO-MIL-100(Fe)	5	THF	rt	47
11	GO-MIL-100(Fe)	5	DMF	rt	27
12	GO-MIL-100(Fe)	5	Urea/ChCl (2:1)	rt	62
13	GO-MIL-100(Fe)	5	Glycerol/ChCl (2:1)	rt	57
**14**	**GO-MIL-100(Fe)**	**5**	**Glycerol/K**_**2**_**CO**_**3**_** (2:1)**	**rt**	91
15	GO-MIL-100(Fe)	10	Glycerol/K_2_CO_3_ (2:1)	rt	71
16	GO-MIL-100(Fe)	20	Glycerol/K_2_CO_3_ (2:1)	rt	73
17	GO-MIL-100(Fe)	30	Glycerol/K_2_CO_3_ (2:1)	rt	72
18	GO-MIL-100(Fe)	40	Glycerol/K_2_CO_3_ (2:1)	rt	72
19	GO-MIL-100(Fe)	5	Glycerol/K_2_CO_3_ (2:1)	50	91
20	GO-MIL-100(Fe)	5	Glycerol/K_2_CO_3_ (2:1)	70	93

According to the TGA analysis, GO-MIL-100(Fe) composite with 38.5/61.5 (w%/w%) ratio of graphene oxide to MIL-100(Fe) was selected as the most efficient catalyst in photocatalytic condensation reaction at r.t. in (Glycerol/K_2_CO_3_ (2:1) (DES) as reaction medium). Significant values are in bold.

In order to narrow down the optimization process even further, the reaction was investigated in various solvents, including EtOH, H_2_O, CH_3_CN, DMF, THF, and n-hexane (Table 2, Entries 6–11). However, all these solvents were inappropriate for this reaction. Thus, the model reaction was performed in various DESs (Table 2, Entries 12–14). It was found that the mixture of Glycerol/K_2_CO_3_ (2:1) led to the highest yield compared to other DESs usually seen in the chemical literature. By investigating different amounts of the catalyst, it was found that 5 mg was the closest optimum amount of added catalyst to the actual number (Table 2, Entry 5); though the higher amount of catalyst was also individually surveyed, and showed that the higher mass of catalyst up to 40 mg had no positive effect on the fact that how efficient the referred reaction progresses (Table 2, Entries 15–18), which ultimately brought us back to the earlier optimal mass, 5 mg. As expected of various radical-based oxidative conditions, proven by experiments, there is the issue of oxidant that might have exceeded a threshold level, resulting in an over-oxidation stage. If the amount of the used catalyst (GO-MIL-100(Fe)) goes more than a threshold stage (shown in Entries 15–18), there might be an over-oxidation reaction, leading to by-products such as carboxylic acids. However, as we were focused on the yield of the desired products and have not studied the by-products.

The final step of yield maximization took place around the temperature of the reaction, the model reaction was performed at different temperatures, and the highest yield of desired (dehydrated) product, 2-benzylidenemalononitrile, was obtained at room temperature (r.t.) (Table 2, Entries 19–20).

The introduction of the effect of light is the last station for this work that was analyzed. The photocatalytic activity of the synthesized composite was screened on various benzyl alcohol derivatives, which have been outlined in Table 3 (Under the given condition). Indeed, both electron donors and electron-withdrawing derivatives were employed, and above 85% yields were obtained after 420 min (= 7 h). As can be seen in Table 3, electron-withdrawing groups such as –NO_2_ (or partially –Cl) provide much more product (Table 3, Entry 4a), whereas, for benzyl alcohols, in which an electron-pumping functional group is attached to its aromatic ring, related products with lower yields resulted (Table 3, Entries 1a, 2a, 3a, and 5a). Generally, the more electron can be found at the benzylic site, the faster it proceeds.

**Table 3:** Light-excited preparation of various 2-benzylidenemalononitrile derivatives in the presence of GO-MIL-100(Fe).


Assigned name	**1a**	**2a**	**3a**	**4a**	**5a**
Product*					
Yield (%)	97	90	93	98	89

*Reaction conditions: Substrates (1 mmol), Glycerol/K_2_CO_3_ (2:1) (5 mL), 5 mg of the catalyst at room temperature under light irradiation for 420 min.

Knowing more about the practical elements, having shown their importance in photocatalytic-oxidative transformations, is done in continue.

**Figure 5:**
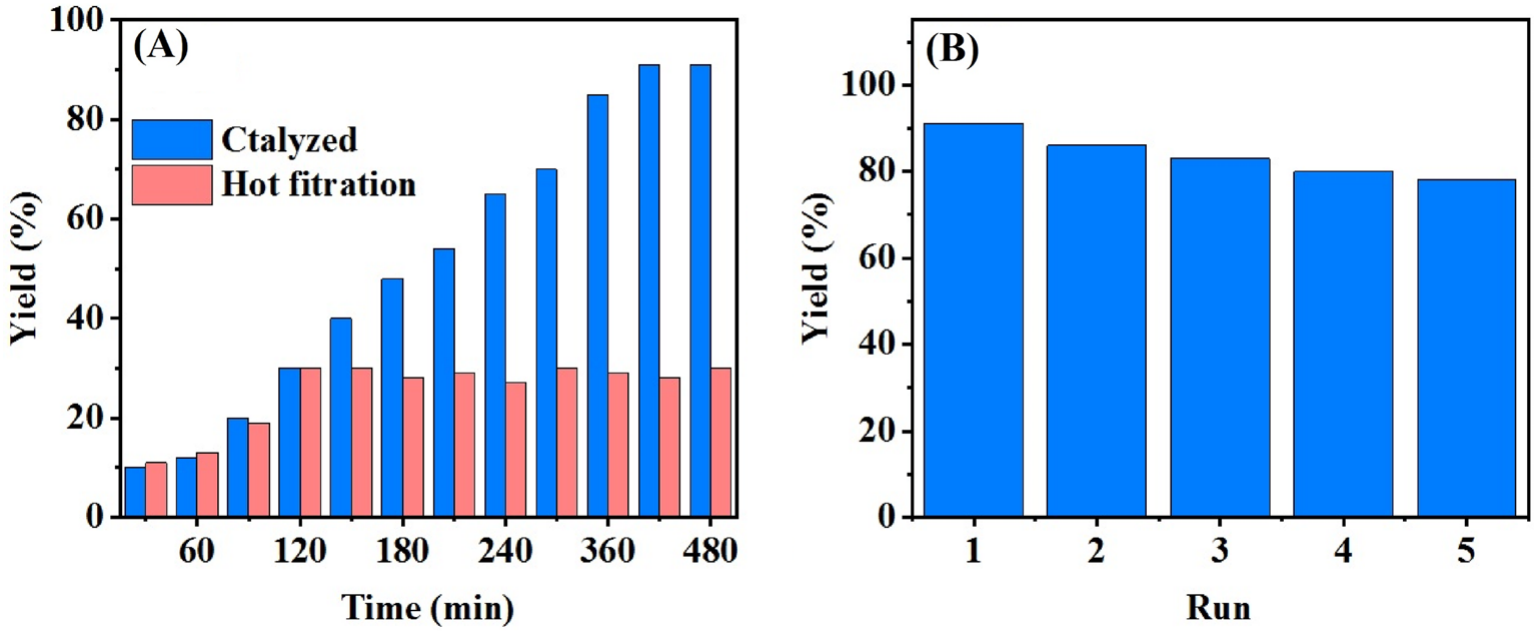
Heterogeneity test (**A**) and catalyst reusability (**B**).

Since the GO-MIL-100(Fe) composite has been generally admitted to be a heterogeneous catalyst, a hot filtration test was utilized to determine the rate of heterogeneity. In this way, the PAOF was performed catalysis by the prepared composite. Then, after 100 min, the reaction continued without the catalyst, and the efficiency of the reaction was controlled by GC-FID. From then on, no changes in the yield of reaction were observed, as if no catalyst leakage was there in the reaction mixture. Therefore, it was concluded that the GO-MIL-100(Fe) is totally heterogeneous and is appropriate enough to undergo the norm of the PAOF (Fig. 5A).

After ensuring the heterogeneity of GO-MIL-100(Fe), its stability and reusability were studied. First of all, after the first run of reaction, we did the catalyst re-gathering by centrifugation, washing with ethanol, and drying in an oven at 80 °C. Next, this recovered solid catalyst was added to the reaction medium in the second run. This experiment passed via five repetitions, and the developed composite was quite efficient after the 5th run (Fig. 5B).

**Figure 6:**
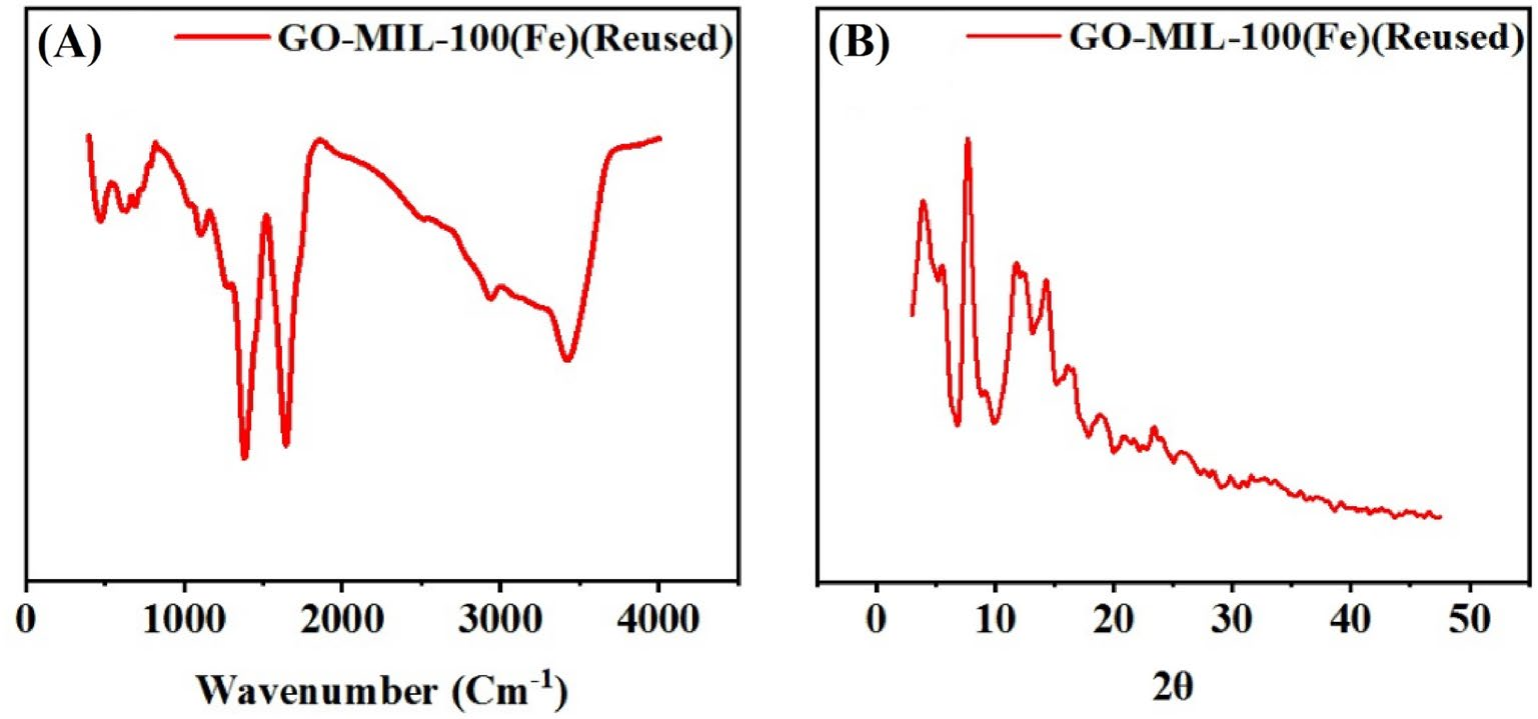
FT-IR (**A**) and PXRD (**B**) spectra of the recovered heterogeneous catalyst after 5th run.

To confirm our hypothesis, the obtained retrieved catalyst from the 5th cycle was analyzed by the FT-IR and PXRD methods, and as Fig. 6A,B displays, the obtained FT-IR spectrum and XRD pattern were almost like the ones that had already synthesized, confirming the high integrity and stability of GO-MIL-100(Fe) nanocomposite.

### Prediction of mechanism using observations and mathematical equations

Having observed the physico-chemical levers of this PAOF reaction, theoretically, the valence band (VB) and the conduction band (CB) levels of the MIL-100(Fe) were obtained using the following formulas^49^:

1 $${\text{E}}_{{{\text{VB}}}} = \, \upchi \, - {\text{ E}}_{{\text{e}}} + \, 0.5{\text{E}}_{{\text{g}}}$$

2 $${\text{E}}_{{{\text{CB}}}} = {\text{ E}}_{{{\text{VB}}}} - {\text{ E}}_{{\text{g}}}$$

In these equations, E_VB_ and E_CB_ are the VB and CB potentials, respectively. E_e_ is the energy of free electrons, and E_g_ is the band gap energy of the semiconductor, which is 3.19 eV for MIL-100(Fe). The last parameter shown as χ is the electronegativity of any semiconductor, which is obtained through the equation below:

3 $${\rm X} = \left[ {\upchi \left( {\text{A}} \right){\text{a}}\upchi \left( {\text{B}} \right){\text{b}}\upchi \left( {\text{C}} \right){\text{c}}} \right]{1}/\left( {{\text{a}} + {\text{b}} + {\text{c}}} \right)$$

**Figure 7:**
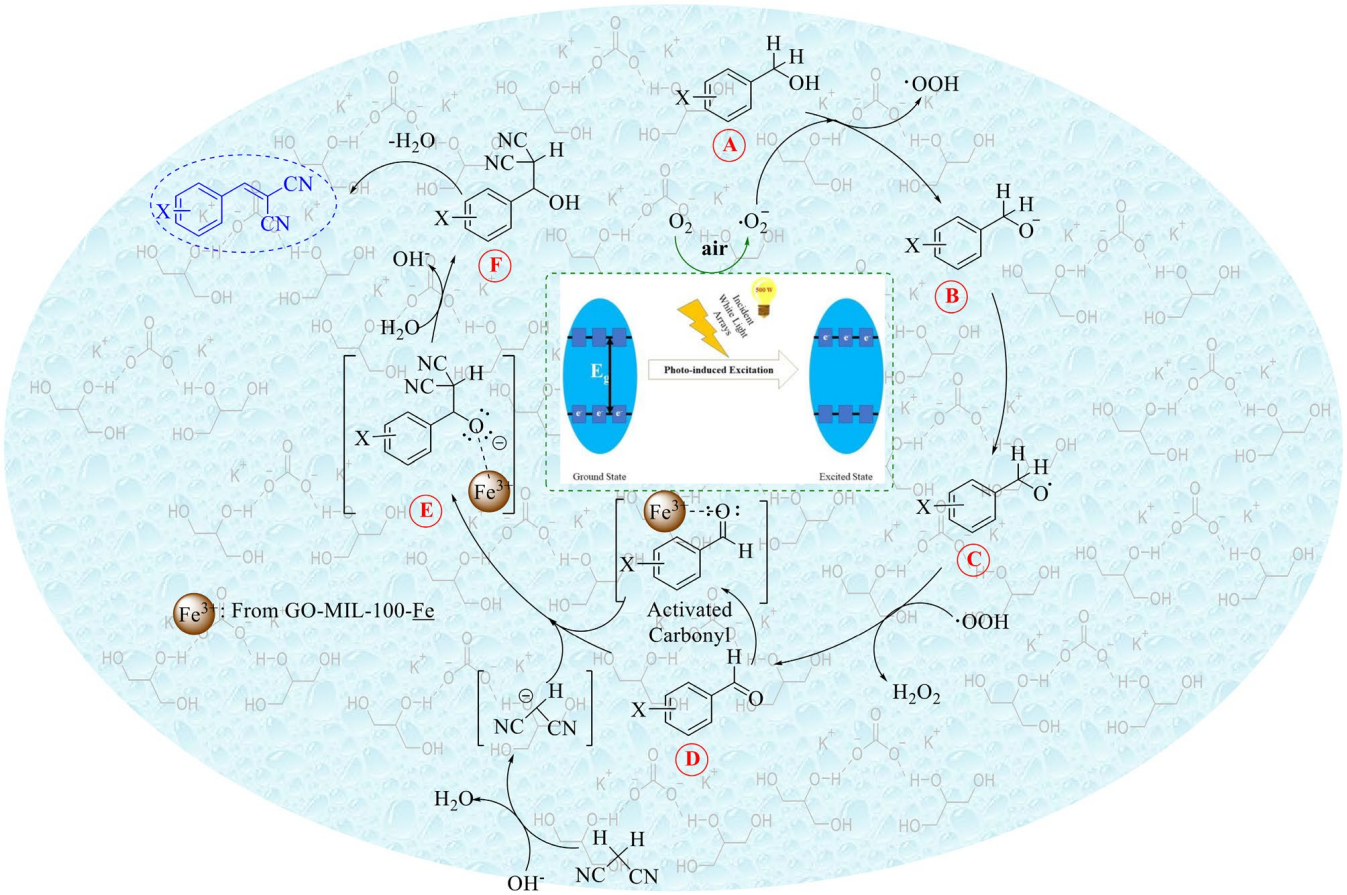
Proposed mechanism for the current PAOF.

In which a, b, and c stand for the number of atoms in the compounds^50^. The χ value for MIL-100(Fe) is obtained equal to 4.64 eV. Thus, the E_CB_ and E_VB_ of MIL-100(Fe) were calculated at about − 0.43 and 2.76 eV. Summarization of the obtained results directed us to a plausible mechanism of condensation reaction has been illustrated in Fig. 7. As shown in this figure, initially, since MIL-100(Fe) has more negative CB (− 1.23 eV vs. NHE) than the level of O_2_/O_2_^**·**−^ (− 0.33 eV vs. NHE), the photoexcited electrons on the CB of MIL-100(Fe) are transferred to the conjugated system of graphene oxide and convert adsorbed O_2_ molecules to O_2_^**·**−^ radicals. This charge transfer disrupts the recombination of these photo-induced electron–hole pairs, leading to a diverge stance and increasing the photocatalytic activity of the utilized catalyst. It is worth mentioning that the oxidation of alcohols is not carried out by the produced holes in the VB of MIL-100(Fe). As Reported by a noticeable paper in the field of MOFs, it has shown photocatalytic activities with the aid of charge separation and reactant activation. So, this modification of the surface of GO sheets enabled us to promote it by adding MIL-100(Fe)^51^. Given in Fig. 7, the oxidation reaction has expected to meet molecular oxygen radical-anion (superoxide; O_2_^**·**−^) and its protonated isologue at one step (A to B). The primary alcohol (benzyl alcohol) loses its proton existing in the hydroxyl (O–H) site (A to B; by releasing protonated superoxide radical (·O–OH)). Then, the deprotonated alcohol is oxidized by that released ·O–OH to form the desired benzaldehyde (D) (or its derivatives). The final synthesized benzaldehyde will react with malononitrile. Parallel to the deprotonation of the α-CH of malononitrile by K_2_CO_3_^52^, the in situ formed benzaldehyde is also activated by the Fe^3+^ ions of MOF. Both of these activated species react together and form E. By releasing an OH^−^ and protonation, F is obtained. Via dehydration, the desired product can be gained. The cycle frequently repeats up to 5 runs without a sensible decline in the efficiency of the earlier catalyst.

In a simpler way to explain, the resulting aldehyde species reacts with malononitrile, and the expected products will be there.

## Conclusion

In conclusion, the GO-MIL-100(Fe) composites containing MOF nanoparticles loaded on graphene oxide sheets were prepared through a sustainable method and employed as a catalyst in the photocatalytic one-pot synthesis of 2-benzylidenemalononitrile from benzyl alcohols and malononitrile under visible light. This catalytic system has a number of merits, including the green preparation method of the composite, performing the reaction under visible light without using oxidant, and, Glycerol/K_2_CO_3_ (2:1) as the best solvent. Meanwhile, the mechanism of PAOF was investigated using trapping experiments, and O_2_^**·**–^, h^+^ and visible light were determined as key elements for performing such reaction. Moreover, the developed composite showed high heterogeneity and reusability after five cycles. In fact, these positive features can improve the sustainability and stability of this catalytic system and shows its high economic efficiency and recovery in the end when it was put into comparison with formerly-published methods.

## Data Availability

All data generated or analyzed during this study are included in this published article.
